# Genome-wide association analysis of agronomic traits in wheat under drought-stressed and non-stressed conditions

**DOI:** 10.1371/journal.pone.0171692

**Published:** 2017-02-24

**Authors:** Learnmore Mwadzingeni, Hussein Shimelis, D. Jasper G. Rees, Toi J. Tsilo

**Affiliations:** 1 School of Agricultural, Earth and Environmental Sciences, University of KwaZulu-Natal, Pietermaritzburg, South Africa; 2 Agricultural Research Council-Small Grain Institute (ARC-SGI), Bethlehem, South Africa; 3 Agricultural Research Council-Biotechnology Platform, Pretoria, South Africa; Aberystwyth University, UNITED KINGDOM

## Abstract

This study determined the population structure and genome-wide marker-trait association of agronomic traits of wheat for drought-tolerance breeding. Ninety-three diverse bread wheat genotypes were genotyped using the Diversity Arrays Technology sequencing (DArTseq) protocol. The number of days-to-heading (DTH), number of days-to-maturity (DTM), plant height (PHT), spike length (SPL), number of kernels per spike (KPS), thousand kernel weight (TKW) and grain yield (GYLD), assessed under drought-stressed and non-stressed conditions, were considered for the study. Population structure analysis and genome-wide association mapping were undertaken based on 16,383 silico DArTs loci with < 10% missing data. The population evaluated was grouped into nine distinct genetic structures. Inter-chromosomal linkage disequilibrium showed the existence of linkage decay as physical distance increased. A total of 62 significant (*P* < 0.001) marker-trait associations (MTAs) were detected explaining more than 20% of the phenotypic variation observed under both drought-stressed and non-stressed conditions. Significant (*P* < 0.001) MTA event(s) were observed for DTH, PHT, SPL, SPS, and KPS; under both stressed and non-stressed conditions, while additional significant (*P* < 0.05) associations were observed for TKW, DTM and GYLD under non-stressed condition. The MTAs reported in this population could be useful to initiate marker-assisted selection (MAS) and targeted trait introgression of wheat under drought-stressed and non-stressed conditions, and for fine mapping and cloning of the underlying genes and QTL.

## Introduction

Wheat is an important commodity crop and a vital component of a healthy global diet providing starch, dietary proteins, fiber, fats, vitamin B, zinc, calcium, and iron [1]. Its production and productivity in most parts of the world, especially in sub-Saharan Africa, is constrained by drought and high temperature stresses [2–4]. Breeding for drought tolerance is one of the key components that could enhance sustainable wheat production and productivity. Concerted research efforts are underway directed at understanding and dissection of the genetic basis of complex traits including drought tolerance through association analysis of genomic regions and agronomic traits [5–9].

Genome-wide association study (GWAS) facilitates understanding of the genetic bases and dissection of complex genes controlling economic traits such as drought tolerance. Genome-wide association analysis rely on marker-trait association (MTA) involving representative markers and genetically diverse populations such as elite breeding lines and improved cultivars. The goal of GWAS is to discern genomic regions that could either be markers, genes or QTL associated with key agro-morphological traits for marker-assisted breeding, gene discovery or gene introgression [10].

Understanding the population structure and the magnitude of linkage disequilibrium (LD) present in the prevailing genetic resources are important pre-requisites to deduce the genetic makeup, composition and genomic predictions of traits of interest during selection. Linkage disequilibrium *per se* could serves as a predictor of the resolution at which influential genomic regions can be detected through marker-trait-association analysis. Linkage analysis establishes associations among sets of genes, and provides insights on the effect of genetic drift, selection, mutation, recombination, quantitative trait loci, linked genes, or gene-flow in a given population [11, 12]. Identification of diagnostic genetic markers, candidate genes and QTL associated with target traits will facilitate marker-assisted selection, and trait introgression. Bread wheat (*Triticum aestivum* L., 2n = 6x = 42; AABBDD) has a genome size of ≈ 17 giga base pairs [13]. A considerable number of markers, genes and/or QTL associated with several polygenic traits has been mapped along the 21 chromosomes of bread wheat [10, 14–17]. These genomic resources are crucial to understand the genetic mechanism of drought tolerance and other economic traits present in complex polyploid crops including wheat.

Several DNA-based marker systems have been successfully applied in association mapping of complex traits in different crop species. The most widely used marker systems include simple sequence repeat (SSR), amplified fragment length polymorphism (AFLP), single nucleotide polymorphism (SNP) and microarray based Diversity Arrays Technology (DArT) markers [18–21]. Advanced and high-throughput genotyping technologies such as genotyping by sequencing (GBS) are effective tools to detect abundant and highly reproducible SNPs and DArT markers [22, 23]. These marker systems are used in population genetics, GWAS, marker assisted selection (MAS), genomic selection, haplotype mapping, genetic diversity analyses or linkage map construction [24]. The Diversity Arrays Technology has been successfully used in wheat, though it was initially developed for crops with less complex genomes such as rice [20, 25, 26, 27, 28]. The DArT sequencing platform provides a database of sequences which are useful resources to advance marker-trait association analyses.

A diverse population of drought and heat tolerant lines were acquired from the International Maize and Wheat Improvement Center (CIMMYT) for selection and drought tolerance breeding in South Africa. The population comprising of 87 introductions and six local drought-susceptible released varieties were screened using key agronomic traits and proline analyses under drought-stressed and non-stressed conditions [29]. Evaluated traits showed moderate to high heritability estimates (Table 1). This provided a comprehensive database of agronomic traits useful for further selection and to undertake marker-trait association analysis. Further, these genetic resources should be systematically genotyped using a fairly large marker density representing the 21 chromosomes to identify additional candidate genes and quantitative trait loci (QTL) controlling key traits to initiate marker-assisted selection of drought tolerance in wheat. Therefore, the objective of this study was to determine the population structure and genome-wide marker-trait association of key agronomic traits of wheat for drought-tolerance breeding, using representative DArTseq markers.

**Table 1:** Broad sense heritability estimates for nine phenotypic traits of 96 wheat genotypes evaluated in two localities over two seasons, under two water regimes and two replications.

Parameter	Traits								
	DTH	DTM	PH	TN	SL	SPS	KPS	TWS	GY
Heritability (H^2^)	0.76	0.47	0.86	0.29	0.95	0.87	0.78	0.68	0.39
Heritability (%)	76.26	47.29	86.33	28.83	94.61	87.28	78.43	68.15	38.93

DTH days to 50% heading, PH plant height, TN number of productive tillers, DTM days to maturity, SL spike length, SPS number of spikelets per spike, KPS number of kennels per plant; TSW thousand seed weight, GY gain yield.

## Methods

### Plant materials and phenotyping

The study used a population of 93 bread wheat genotypes that were purposefully selected for drought tolerance breeding, based on their divergent pedigree and reaction to drought stress. This sample size was in the same range with those successfully used in some previous studies [18, 27, 28, 30]. Eighty-seven lines were included from a set of heat and drought tolerant and susceptible nurseries received from CIMMYT, while the remaining six lines were drought-susceptible local checks widely grown in South Africa. The 93 genotypes were rigorously phenotyped for key agronomic traits across eight testing environments involving two cultivations (greenhouse and field), two seasons (2014/2015 and 2015/2016) and two contrasting water regimes (drought-stressed and non-watered conditions) [29]. The population was phenotyped for the number of days-to-heading (DTH), plant height (PHT), number of days-to-maturity (DTM), spike length (SPL), number of spikelets per spike (SPS), numbers of kennels per spike (KPS), thousand kernel weight (TKW) and grain yield per plot (GYLD). The data were checked for normality, homogeneity of variance and validity for analysis of variance following the Bartlet (1974) test [31], and were then analyzed as described by Mwadzingeni et al. [29]. Broad sense heritability (H^2^) estimates were calculated from phenotypic variance (σ^2^_*p*_) and the genotypic variance (σ^2^_*g*_) according to Allard [32] using the formula;

$${\text{H}}^{2}=\sigma^{2}{}_{g}/\;(\sigma^{2}{}_{g}+\sigma^{2}{}_{gwls}/wls+\sigma^{2}{}_{gls}/ls+\sigma^{2}{}_{glw}/lw+\sigma^{2}{}_{gsw}/sw+\sigma^{2}{}_{gs}/s+\sigma^{2}{}_{gw}/w+\sigma^{2}{}_{gl}/l+\sigma^{2}{}_{e}/rlsw)\;=\sigma^{2}{}_{g}/\;(\sigma^{2}{}_{g}+\sigma^{2}{{}_{g}}_{xe}/e+\sigma^{2}{}_{e}/re)=\sigma^{2}{}_{g}/\sigma^{2}{}_{p}.$$

Where *w*, *l*, *s* and *r* were the water regime, site, season and replications respectively.

### DNA extraction and DArT sequencing

Genomic DNA of the 93 genotypes was extracted from fresh leaf tissue of 2 week old seedlings following the plant DNA extraction protocol for DArT [33]. The quality of DNA was checked for nucleic acid concentration and purity using a NanoDrop 2000 spectrophotometer (ND-2000 V3.5, NanoDrop Technologies, Inc.). The DNA samples were sent to Diversity Arrays Technology Pty Ltd, Canberra, Australia in a 96 well microtiter plate for destructive DNA analysis. Samples were genotyped using the DArTseq protocol using 38,611 silico DArTs. After eliminating the DArT loci with unknown chromosome positions and filtering markers with more than 10% missing data, a total of 16,383 markers distributed across the 21 chromosomes were maintained for analysis. The number of markers used from each chromosome were 681; 1,068; 289; 1,114; 1,887; 455; 754; 1,322; 396; 995; 334; 86; 512; 1,242; 150; 868; 1,303; 264; 1,145; 1,231 and 287 in chromosomes 1A, 1B, 1D, 2A, 2B, 2D, 3A, 3B, 3D, 4A, 4B, 4D, 5A, 5B, 5D, 6A, 6B, 6D, 7A, 7B and 7D, in that order. The silico DArTs used had reproducibility values of 1, polymorphic information content (PIC) values ranging from 0.02 to 0.50, a mean call rate of 0.93 with a range from 0.84 to 1, and a read mean depth of 14.92 ranging from 5 to 399.

### Population structure, linkage disequilibrium and marker-trait association analyses

Population structure was determined using the software STRUCTURE v2.3.4 [34]. The parameters of the project were set at 10,000 burn-in periods, with 10,000 Markov chain–Monte Carlo (MCMC) repetitions after burn-in. Ten iterations were ran for K values of 1 to 20 to allow selection of the replication with the highest mean value of ln likelihood. Genotypic data was imputed for missing values using TASSEL v4.3.15 (https://sourceforge.net/p/tassel/tassel4-standalone/ci/master/tree/). Linkage disequilibrium was estimated using the squared allele frequency correlations R^2^ value from which the number of significant allele pairs (*P* < 0.01) was determined using 1,000 permutations. Marker trait association analysis, probability values and % of the effect of these markers were calculated following the GLM procedure in TASSEL v4.3.15 according to Bradbury et al. [35].

## Results

### Phenotypic traits evaluation

Analysis of variance indicated significant differences (*P* < 0.05) due to the genotype, site, water regime and their interaction effects for all the studied traits. These and the Pearson’s correlations (*r*) were reported by Mwadzingeni et al. [29]. High and positive correlations and high heritability estimates were detected for most of the traits considered in the current study. Spike length, number of spikelets per spike, plant height, number of kennels per spike, number of days-to-heading and thousand kernel weight had higher levels of genotypic variance (σ^2^_*g*_), hence high heritability values of > 50% (Table 1). The number of days-to-maturity and grain yield had moderate heritability estimates (20% ≤ H^2^ < 50%).

### Population structure

**Fig 1:**
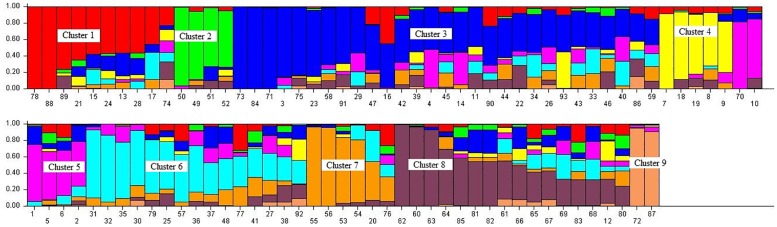
Population structure based on 93 genotypes and 16,383 DArTseq markers. Each colored segment per genotype estimates the membership fraction to each of the 9 populations. See Table 1 for codes of genotypes.

Population structure was constructed to reveal the genetic relationships and to aid genotype selection. Nine distinct populations were recognised (Fig 1) after the LnP (D) kept increasing from -766,307 at K = 1 to -627,026 (with a mean value of ln likelihood of -590,791) at K = 9. Fig 1 presents the population structure for K = 9 where each colour represents a different genetic cluster. The list of genotypes and the overall representation of membership of the sample in each of the 9 clusters are presented in Table 2. The expected heterozygosity of genes among individuals varied from 0.07 to 0.29 with fixation index (Fst) varying from 0.31 to 0.89 among clusters.

**Table 2:** Nine genetic clusters with their respective list of wheat genotypes, proportion of membership, expected heterozygosity and the mean values of Fst observed from the study population.

Cluster	*Genotypes	% membership	Expected heterozygosity	Mean fixation index (Fst)
1	LM13 (13), LM15 (15), LM17 (17), LM21 (21), LM25 (24), LM29 (28), LM80 (74), LM84 (78), LM95 (88), LM96 (89)	0.118	0.1682	0.6144
2	LM50 (49), LM51 (50), LM52 (51), LM53 (52)	0.06	0.0908	0.8375
3	LM03 (3), LM04 (4), LM11 (11), LM14 (14), LM16 (16), LM22 (22), LM24 (23), LM27 (26), LM30 (29), LM34 (33), LM35 (34), LM40 (39), LM41 (40), LM43 (42), LM44 (43), LM45 (44), LM46 (45), LM47 (46), LM48 (47), LM59 (58), LM60 (59), LM77 (71), LM79 (73), LM81 (75), LM90 (84), LM93 (86), LM97 (90), LM98 (91), LM100 (93)	0.235	0.171	0.561
4	LM07 (7), LM08 (8), LM09 (9), LM18 (18), LM19 (19)	0.073	0.0674	0.892
5	LM01 (1), LM02 (2), LM05 (5), LM06 (6), LM10 (10), LM76 (70)	0.1	0.0863	0.8545
6	LM26 (25), LM28 (27), LM31 (30), LM32 (31), LM33 (32), LM36 (35), LM37 (36), LM38 (37), LM39 (38), LM42 (41), LM49 (48), LM58 (57), LM83 (77), LM85 (79), LM99 (92)	0.142	0.2472	0.441
7	LM20 (20), LM54 (53), LM56 (55), LM57 (56), LM82 (76)	0.102	0.1093	0.7725
8	LM12 (12), LM61 (60), LM64 (61), LM66 (62), LM67 (63), LM68 (64), LM70 (65), LM71 (66), LM72 (67), LM73 (68), LM75 (69), LM86 (80), LM87 (81), LM88 (82), LM89 (83), LM91 (85)	0.136	0.2947	0.3117
9	LM78 (72), LM94 (87)	0.033	0.1027	0.8471

See Mwadzingeni et al. [29] for genotype pedigrees.

In the structure, Cluster 1 consisted of six and four genotypes from the heat and drought tolerance nurseries, respectively (Table 2). Cluster 2 consisted of only four genotypes from the heat tolerance nursery. This was followed by the largest group (Cluster 3) which comprised of 29 genotypes of which 21 were from the heat tolerance nursery while the remaining eight were from the drought tolerance nursery. Cluster 4 had only genotypes from the heat tolerance nurseries, while Clusters 5, 6 and 7 had mixtures of genotypes. All the local checks (LM61, LM64, LM66, LM67 and LM70) were grouped in Cluster 8, together with ten other genotypes including LM12 from the heat tolerance nursery and nine genotypes from the drought tolerance nursery (Table 2). Likewise, the last cluster contained the genotypes LM78 and LM94 from the drought tolerance nursery.

### Linkage disequilibrium

Linkage disequilibrium analysis revealed the presence of 597,871 loci pairs within a physical distance extending up to 16,356 bp. About 45,835 (7.67%) of loci pairs were in significant LD (*P* < 0.05). Further, 5,188 (0.87%) of the pairs were in complete LD (R^2^ = 1). Marker pairs in LD were observed over long distances, however, a clear and rapid decline in LD with distance was observed. Pearson’s correlation coefficients revealed negative correlation (*r* = -0.0813 between the linkage disequilibrium (R^2^) and the physical distance (bp); as well as between the P-value and R^2^ (*r* = -0.59), revealing the existence of linkage decay.

### Marker-trait association

A total of 334 significant (*P* < 0.05) marker-trait associations (MTAs) were observed. S1 Table provides the list of significant (0.05 > *P* > 0.001) MTAs that could also have influence on respective traits. Only the MTAs that had *P* values < 0.001 (Table 3) were considered as significant for all traits except for grain yield, thousand seed weight and number of days-to-maturity where significant (*P* < 0.05) marker-trait associations were considered because the three traits are highly complex, often with moderate to low heritability [36]. These markers explained > 20% of the total phenotypic variation observed on all respective traits. Of the MTAs that were considered significant, four loci were identified to be highly associated with the number of days-to-heading, explaining 24.96% to 37.77% of the total phenotypic variation. Two of these makers were located on chromosome 5A, while the other two were found on chromosomes 5B and 6B (Table 3). The number of days-to-heading were recorded immediately before imposing drought stress but the means from the stressed and non-stressed experiments were used separately for GWAS to check for repeatability.

**Table 3:** DArTseq markers with high association with eight agronomic traits of 93 wheat genotypes evaluated under drought-stressed and non-stressed conditions.

Trait	Drought-stressed			Non-stressed		
	Marker	Perm P	Marker R^2^	Marker	Perm P	Marker R^2^
**Days-to-heading**	5A|081.525617550|4542594|4542594	0.001	0.31411	5A|081.525617550|4542594|4542594	0.001	0.29551
	5A|084.411633690|3534155|3534155	0.001	0.37532	5A|084.411633690|3534155|3534155	0.001	0.37773
	5B|000.649324338|1209883|1209883	0.001	0.24964	5B|000.649324338|1209883|1209883	0.001	0.24964
	6B|079.586479380|3949288|3949288	0.001	0.30387	6B|079.586479380|3949288|3949288	0.001	0.30809
**Plant height**	1B|063.445873190|3937163|3937163	0.001	0.28054	2B|013.546408570|977308|977308	0.001	0.28426
	2D|128.146584600|4021827|4021827	0.001	0.28848	2B|023.182120080|1251215|1251215	0.001	0.26467
				5A|084.411633690|3534155|3534155	0.001	0.2667
				5B|000.649324338|1209883|1209883	0.001	0.23751
				6B|079.586479380|3949288|3949288	0.001	0.25864
				7B|112.004439500|2322338|2322338	0.001	0.26883
**Spike length**	2B|107.092980900|1029432|1029432	0.001	0.22172	2B|107.092980900|1029432|1029432	0.001	0.21201
	2B|108.086871100|1132117|1132117	0.001	0.22172	2B|108.086871100|1132117|1132117	0.001	0.21201
	2D|128.146584600|4021827|4021827	0.001	0.3196	2D|128.146584600|4021827|4021827	0.001	0.30445
	5B|000.000000000|3023157|3023157	0.001	0.24953	5B|000.000000000|3023157|3023157	0.001	0.24497
	5B|117.097644100|3950938|3950938	0.001	0.22692	5B|117.097644100|3950938|3950938	0.001	0.22025
	6B|079.586479380|3949288|3949288	0.001	0.27346	6B|079.586479380|3949288|3949288	0.001	0.27701
	7A|065.934336980|1118335|1118335	0.001	0.25871	7A|065.934336980|1118335|1118335	0.001	0.24691
	3A|056.634055130|3934533|3934533	0.001	0.23714	6B|031.043100140|1237876|1237876	0.001	0.20889
	1B|063.445873190|3937163|3937163	0.001	0.22512			
	1B|184.429245100|1113389|1113389	0.001	0.22723			
	4B|042.180040830|1081624|1081624	0.001	0.22838			
	4B|042.901192180|1027953|1027953	0.001	0.22838			
	6A|048.638907120|3944784|3944784	0.001	0.23035			
**Days-to-maturity**				6B|079.586479380|3949288|3949288	0.012	0.24028
**Spikelets per spike**	1B|239.642526900|1249348|1249348	0.001	0.33723	1B|239.642526900|1249348|1249348	0.001	0.32517
	2D|128.146584600|4021827|4021827	0.001	0.38306	2D|128.146584600|4021827|4021827	0.001	0.35446
	4B|042.180040830|1081624|1081624	0.001	0.32331	4B|042.180040830|1081624|1081624	0.001	0.33309
	4B|042.901192180|1027953|1027953	0.001	0.32331	4B|042.901192180|1027953|1027953	0.001	0.33309
	5B|000.649324338|1209883|1209883	0.001	0.31848	5B|000.649324338|1209883|1209883	0.001	0.28063
	6B|079.586479380|3949288|3949288	0.001	0.4069	6B|079.586479380|3949288|3949288	0.001	0.36461
	5B|000.000000000|3023157|3023157	0.001	0.34661	5A|084.411633690|3534155|3534155	0.001	0.32471
	2B|107.007987900|1087177|1087177	0.001	0.38011			
**Kernels per spike**	2D|128.146584600|4021827|4021827	0.001	0.30042	2D|128.146584600|4021827|4021827	0.001	0.30059
	4A|132.059989400|4989948|4989948	0.001	0.30242	2D|148.989565100|374614|wPt-4329	0.001	0.2447
				6B|031.043100140|1237876|1237876	0.001	0.29653
				6B|031.199477690|4990947|4990947	0.001	0.34092
				6B|033.035441000|1300029|1300029	0.001	0.34092
				6B|035.712467660|4989379|4989379	0.001	0.28856
				7A|048.712205470|1129617|1129617	0.001	0.28859
				7A|065.934336980|1118335|1118335	0.001	0.29523
**1,000 seed weight**				7B|076.034196290|1258792|1258792	0.03	0.23944
**Grain yield**				5D|138.209637900|7157166|7157166	0.021	0.22568

Perm P = Probability value; R^2^ = marker-trait correlation

Marker-trait- association analyses revealed association between specific phenotypes and genetic variants within a genome, which could lead to the discovery of genes controlling the traits. Two markers located on chromosomes 1A and 2D were associated with plant height under drought-stress. Under non-stressed condition, six markers were associated with plant height of which two were located on chromosome 2B and the rest were on chromosomes 5A, 5B, 6B, and 7B. These markers explained 23.75% to 28.8% of the variation in plant height. Spike length was associated with thirteen markers under drought-stressed condition explaining 22.17% to 31.96% of the total phenotypic variation; and eight markers under non-stressed condition; explaining 21.20% to 30.45% of the variation in spike length. The markers observed for this trait under drought-stress were from chromosomes 1B, 2B, 2D, 3A, 4B, 5B, 6A, 6B and 7A. Eight DArT markers were associated with spike length under non-stressed condition of which seven markers were consistent under both drought-stressed and non-stressed conditions from chromosomes 2B, 2D, 5B, 5B and 7A (Table 3). Under drought-stress, SPS was highly associated with eight markers located on chromosomes 6B, 2D, 2B, 5D, 1B and 4B; while under the same stress level, seven significant MTAs were recorded that were located on chromosomes 1B, 2D, 4B, 5A, 5B and 6B. Six of the markers, except for one located on chromosome 2B, one on 5A and one on 5B were consistent with the ones obtained under drought-stressed condition (Table 3).

The B genome had most of the significant MTAs observed for this trait. The marker 6B|079.586479380|3949288|3949288 explained the highest proportion of the phenotypic variation (41%) under drought-stressed condition while a marker on chromosome 2B explained the least proportion (28.06%) of the phenotypic variation observed under the non-stressed condition. Under drought-stressed condition, the number of kernels per spike was associated with two markers located on chromosomes 2D and 4A, explaining 30.04% and 30.24% of the observed phenotypic variation, in that order. Eight significant MTAs were detected under non-stressed condition on chromosomes 2D, 6B and 7A explaining 28.06% to 36.46% of the variation of the number of spikelets per spike, respectively. Three MTAs on chromosomes 6B, 7B and 5D were considered significant (0.05 > *P* > 0.001) for the number of days-to-maturity, thousand seed weight and grain yield accounting for 24.03%, 23.94% and 22.57% of the phenotypic variation, respectively. Table 4 summarises the number of DArTseq markers observed for each of the nine agronomic traits evaluated under drought stressed and non-stressed conditions. Subject to further validation, these markers will be useful for marker-assisted selection for respective traits under target growing conditions.

**Table 4:** Number of DArTseq markers with high association with nine agronomic traits evaluated under drought stressed and non-stressed conditions.

Trait	Drought-stressed		Non-stressed	
	Number of MTAs	Chromosomes	Number of MTAs	Chromosomes
Days-to-heading	4	5A(2), 5B, 6B	4	5A(2), 5B, 6B
Plant height	2	1B, 2D	6	2B, 5A, 5B, 6B, 7B
Spike length	13	1B(2), 2B(2), 2D, 3A, 4B(2), 5B(2), 6A, 6B, 7A	8	2B, 2D, 5B, 6B, 6B, 7A
Days-to-maturity	0	-	1	6B
Spikelets per spike	8	1B, 2B, 2D, 4B(2), 5B(2), 6B	7	1B, 2D, 4B, 5A, 5B, 5D, 6B
Kernels per spike	2	2D, 4A	8	2D(2), 6B(4), 7A(2), 7B
1000 Seed weight	0	-	1	7B
Grain yield	0	-	1	5D
Tiller numbers	0	-	0	-

Brackets enclose the number of MTA events observed only if more than one.

A pleiotropic locus is associated and affects the expression of more than one phenotypic trait. In this study, several pleiotropic loci were identified including the marker 5A|084.411633690|3534155|3534155 that was associated with DTH, PHT and SPS under non-stressed condition (Table 3). Days-to-heading, PHT and DTM under non-stressed condition; SPL under drought-stressed condition; and SPS under drought-stressed condition were associated with the marker 6B|079.586479380|3949288|3949288 located on chromosome 6B. On chromosome 2D, the locus 2D|128.146584600|4021827|4021827 was associated with PHT under drought-stress condition as well as SPL, SPS, and KPS under both drought-stressed and non-stressed conditions. Plant height and SPL under drought-stressed condition were associated with the marker 1B|063.445873190|3937163|3937163 on chromosome 1B. The marker 7A|065.934336980|1118335|1118335 was associated with SPL under both drought-stressed and non-stressed conditions as well as with KPS under non-stressed condition. Additionally, 5B|000.000000000|3023157|3023157 was associated with SPL under drought-stressed and non-stressed conditions as well as with SPS under drought-stressed condition only. Spike length and SPS under drought-stressed condition were associated with the marker 2B|108.086871100|1132117|1132117, while the marker 4B|042.180040830|1081624|1081624 was associated with SPL under drought-stressed condition only and SPS under both drought-stressed and non-stressed conditions. Further, the locus 5B|000.649324338|1209883|1209883 was associated with DTH and PHT under non-stressed condition as well as SPS under drought-stressed condition. Finally, 6B|031.043100140|1237876|1237876 was associated with SPL and KPS under drought-stressed condition. Blast searches of the marker 6B|031.043100140|1237876|1237876 on the National Center for Biotechnology Information (NCBI) (http://blast.ncbi.nlm.nih.gov/Blast.cgi) and GrainGenes (http://wheat.pw.usda.gov/GG2/blast.shtml) databases indicated that this marker has a sequence alignment that is 97% identical to the TaMFT gene that regulates seed dormancy on chromosome 3A (Nakamura et al. [37]; http://www.uniprot.org/uniprot/A0A0K2RW47).

Out of the 65 significant marker-trait associations observed, 25 trait-specific MTAs were differentiated. Chromosome 2B had four trait specific MTAs of which one was associated with spike length under drought-stress, two with plant height under non-stressed condition and one with spike length under non-stressed condition. Traits that were represented by at least one significant trait-specific marker-trait association under either of the two water conditions were days-to-heading, plant height, spike length, number of spikelet per spike, number of kernels per spike, days-to-maturity, and grain yield (Table 3).

## Discussion

Understanding the genetic bases of complex traits in polyploid crops such as wheat, presents an opportunity for drought tolerance breeding. To complement the growing need for such knowledge, the current study explored the population structure and association of genomic regions with yield and yield related traits in a diverse population of drought tolerant and susceptible wheat genotypes. High heritability estimates as well as significant and positive correlations were observed among the studied traits ([29], Table 1) confirming the value of the data in the present marker-trait association analyses. This is supported by Laido et al. [38] who reported the relevance of traits that had high heritability estimates for QTL detection.

### Population structure and linkage disequilibrium

The population evaluated was grouped into nine distinct genetic structures (Fig 1). This is expected given that the genetic materials possess diverse pedigrees which were systematically developed by CIMMYT. However, the existence of common origin or parents in the pedigrees of some genotypes often results in some levels of relationship among genotypes. The results obtained from the structure analysis will be useful in tracking potential parents that could be useful for drought tolerance breeding. Thus, future studies could use a sub-sample of the genetically divergent lines from this genetic pool exhibiting farmers-preferred and quality attributes.

Most of the unique groupings identified could be explained by existence of at least one common parent in the pedigree of genotypes within each cluster. For instance, in Cluster 1, five of the six genotypes from the heat nursery (LM13, LM15, LM21, LM25 and LM29) shared the common parent PASTOR in their parentage. Also, the genotypes LM50, LM51, LM52 and LM53 found in Cluster 2 could be related due to the sharing of crosses involving HUW234+LR34/PRINIA*2// in their parentage. Similarly, the ancestral genotype SOKOLL which was common in most pedigrees of genotypes in Cluster 4 could be the source parent of some similarities in that grouping. Interestingly, all the genotypes in Cluster 5 were descendants from the parents MILAN, KAUZ and PRINIA. Likewise, seven of the genotypes in Cluster 6 had pedigrees containing ATTILA and the parent PBW343 was common in all pedigrees of genotypes in Cluster 7, which could have contributed to formation of these respective clusters.

Existence of marker pairs in LD over long distances and closely linked pairs with non-significant LD observed in the current study has been previously reported in various crop species [27, 30, 39]. This could reflect that LD is not static as it can be influenced by other factors such as genetic admixtures apart from the genetic or physical distance.

### Marker-trait association

The present study identified 334 significant (*P* < 0.05) marker-trait associations (Table 3; S1 Table). This will add to previously identified genomic regions influencing similar or complimentary traits [10, 15, 16]. Although only those MTAs observed at *P* < 0.001 were considered significant in this study, the rest of these associations observed at *P* < 0.05 (S1 Table) may be useful for drought tolerance breeding. These MTAs could be located on regions that influence the respective traits directly or indirectly. Thus, the proportion of the phenotypic variation (R^2^ > 0.2) observed for all significant markers suggests their possible influence on respective traits. In this light, the observed MTAs for grain yield, days to maturity and thousand seed weight, were considered significant at 0.05 > *P* > 0.001, since the traits are highly complex with low heritability [36, 40]. Drought tolerance is highly influenced by genotype by environment interaction [41] which is explained by the lower number of significant MTAs observed under drought-stressed than non-stressed condition.

Several research efforts have been directed at locating genes and QTL influencing various agronomic traits to facilitate MAS in wheat improvement in the face of increased droughts along with other key production constraints [42–44]. The genes identified in the current population adds to the currently available pool of genetic resources and candidate genes. Some of these loci could be located on regions that were already confirmed to be housekeeping genes and QTLs for the traits under study. For instance, in the present study, significant MTAs have been identified on chromosomes that had previously been reported to house QTL for respective traits. Plant height was reported to be associated with genomic regions on chromosomes 1B [44, 45], 2B [42, 44, 45], 5A [45], 5B [44, 45] and 6B [45]. Chromosome 5D was reported to harbour QTL for grain yield [46] in agreement with the present study. Further, Peleg et al. [7] reported loci affecting the number of days-to-heading on chromosome 5A under varied drought-stress levels. In the present study, an MTA for TKW was recorded on Chromosome 7B, which was previously reported to have significant associations with the same trait using the markers *Xwmc606*, *Xgwm537*, *wPt1715* and *wPt2449* in a collection of tetraploid durum wheat genotypes [38]. Blast search on the NCBI database revealed that the DArTseq markers associated with DTH on chromosome 5A in the present study seems to be located on a highly conserved region since it has almost 100% sequence similarities with other regions in other crop species including *Sorghum bicolor* *and* *Oryza sativa* (http://blast.ncbi.nlm.nih.gov/Blast.cgi).

Typically, loci or QTL regions that influence a particular trait under stress also control the trait under non-stressed condition [45]. This could be the case with loci that influenced spike length under non-stressed condition and were consistently observed under drought-stress in the present study. Similar explanation can be presented for the markers affecting the number of spikelet per spike under non-stressed condition that were consistent under drought-stressed condition except for the locus at 5A|084.411633690|3534155|3534155. Ideally, the effects of such loci may not be influenced by the change in external environment. Such genomic regions could be useful in MAS or gene introgression when breeding for broad adaptation. On the other hand, some gene loci may influence particular traits differently under different sets of growing environments, resulting in markers or loci becoming inconsistently associated with particular traits when environmental conditions change. This has been witnessed on markers such as 1B|063.445873190|3937163|3937163 and 4B|042.901192180|1027953|1027953 that were associated with plant height and spike length, respectively, only under drought-stress.

High phenotypic trait correlations could be explained in terms of direct or indirect contribution of one trait to another. Looking into the genome, loci controlling such traits could be similar. This is evidenced by the existence of several multi-trait associations where one gene will have pleotropic effects on highly correlated traits. Dholakia et al. [47] reported that highly correlated traits are often controlled by a common QTL. For instance, the locus 2D|128.146584600|4021827|4021827 controls several traits such as plant height, spike length, number of spikelets per spike and the number of kernels per spike; which are often highly correlated [48, 49]. Such findings support the need to verify if the locus 6B|031.043100140|1237876|1237876 associated with spike length and the number of kernels per spike, is not also linked to seed dormancy, since it shared similar sequence alignment with the region controlling the latter trait in wheat. Interestingly, chromosome 5B which reportedly harbor a region controlling several agronomic traits [10] is found to carry genomic regions associated with DTH, PHT, SPS and SPL in the present study. Some loci, however, influenced only one trait, for instance, 2B|013.546408570|977308|977308 and 2B|023.182120080|1251215|1251215 affected plant height than other traits evaluated in the present study.

## Conclusions

Marker trait association is key to identifying genomic regions that are associated with phenotypic traits of breeding significance. The present study identified a total of 65 highly significant marker trait associations under contrasting water regimes (Table 4). Only one marker per trait was considered significant at *P* = 0.05 for grain yield, days to maturity and thousand seed weight, which had low heritability values. The markers identified in this study are useful genomic resources to initiate marker-assisted selection and trait introgression of wheat under drought-stressed and non-stressed conditions, and for fine mapping and cloning of the underlying genes and QTL. Further studies are required to validate the significant markers identified in the present study using a larger population, following the multiple loci mixed model (MLMM) as proposed by Segura et al. [50] to increase the power of association detection.

## Supporting information

S1 Table DArTseq markers associated with eight agronomic traits of 93 wheat genotypes evaluated under drought-stressed and non-stressed conditions (0.5 > P > 0.001). (DOCX)
