# Supplementary material for: Genome-wide association analysis of agronomic traits in wheat under drought-stressed and non-stressed conditions
Source: PLoS One. 2017 Feb 24;12(2):e0171692. doi: 10.1371/journal.pone.0171692 (PMC5325217; doi:10.1371/journal.pone.0171692)
Supplement: S1 Table — (DOCX) [file pone.0171692.s001.docx]

**DArTseq markers associated with eight agronomic traits at 0.05 > P > 0.001**

| **Drought-stressed** | | | **Non-stressed** | | |
| --- | --- | --- | --- | --- | --- |
| **Marker** | **Perm P** | **Marker R^2^** | **Marker** | **Perm P** | **Marker R^2^** |
| **Days-to-heading** | | | | | |
| 5B\|000.649324338\|1209883\|1209883 | 0.003 | 0.23873 | 2B\|107.007987900\|1087177\|1087177 | 0.002 | 0.29296 |
| 5A\|086.598960660\|1215648\|1215648 | 0.003 | 0.26821 | 2D\|128.146584600\|4021827\|4021827 | 0.003 | 0.26388 |
| 2D\|128.146584600\|4021827\|4021827 | 0.003 | 0.27203 | 5A\|086.598960660\|1215648\|1215648 | 0.007 | 0.2608 |
| 3A\|134.034465000\|3945820\|3945820 | 0.003 | 0.27326 | 1B\|263.548918400\|1396299\|1396299 | 0.009 | 0.28202 |
| 2B\|107.007987900\|1087177\|1087177 | 0.003 | 0.29771 | 4A\|050.549009130\|1118950\|1118950 | 0.014 | 0.21772 |
| 2B\|107.092980900\|1029432\|1029432 | 0.011 | 0.21538 | 6B\|078.748041400\|3958789\|3958789 | 0.024 | 0.26966 |
| 2B\|108.086871100\|1132117\|1132117 | 0.011 | 0.21538 | 3A\|134.034465000\|3945820\|3945820 | 0.026 | 0.24248 |
| 6B\|078.748041400\|3958789\|3958789 | 0.011 | 0.27278 | 2B\|107.092980900\|1029432\|1029432 | 0.034 | 0.20372 |
| 5B\|000.000000000\|3023157\|3023157 | 0.013 | 0.24497 | 2B\|108.086871100\|1132117\|1132117 | 0.034 | 0.20372 |
| 6A\|067.991408960\|1237708\|1237708 | 0.023 | 0.23895 |  |  |  |
| 3B\|013.678476040\|3958709\|3958709 | 0.027 | 0.23445 |  |  |  |
| 3A\|004.294450061\|4990595\|4990595 | 0.028 | 0.23402 |  |  |  |
| 4A\|050.549009130\|1118950\|1118950 | 0.029 | 0.20193 |  |  |  |
| 1B\|037.446808880\|4991863\|4991863 | 0.035 | 0.23014 |  |  |  |
| 6A\|056.724637920\|3945797\|3945797 | 0.043 | 0.19675 |  |  |  |
| 2B\|057.230408490\|3064921\|3064921 | 0.043 | 0.22823 |  |  |  |
| **Plant height** | | | | | |
| 5B\|000.649324338\|1209883\|1209883 | 0.002 | 0.24165 | 3A\|076.019683050\|4394549\|4394549 | 0.002 | 0.22396 |
| 3A\|070.942486970\|1104502\|1104502 | 0.002 | 0.24277 | 1B\|063.445873190\|3937163\|3937163 | 0.002 | 0.25054 |
| 3B\|008.294356567\|4261111\|4261111 | 0.002 | 0.29407 | 3B\|008.294356567\|4261111\|4261111 | 0.002 | 0.27192 |
| 3B\|012.621927070\|3028387\|3028387 | 0.003 | 0.24898 | 3B\|005.715696741\|1125891\|1125891 | 0.003 | 0.24427 |
| 7B\|112.004439500\|2322338\|2322338 | 0.008 | 0.24533 | 3A\|070.942486970\|1104502\|1104502 | 0.004 | 0.21477 |
| 3A\|076.019683050\|4394549\|4394549 | 0.01 | 0.2112 | 4A\|050.549009130\|1118950\|1118950 | 0.009 | 0.20701 |
| 3B\|011.134281090\|3027768\|3027768 | 0.017 | 0.25656 | 2D\|128.146584600\|4021827\|4021827 | 0.013 | 0.22945 |
| 2D\|126.901211300\|1109826\|1109826 | 0.018 | 0.20185 | 3A\|134.034465000\|3945820\|3945820 | 0.013 | 0.22981 |
| 3B\|005.715696741\|1125891\|1125891 | 0.018 | 0.23024 | 3B\|017.067952280\|998573\|998573 | 0.015 | 0.24932 |
| 3B\|013.678476040\|3958709\|3958709 | 0.018 | 0.23051 | 2B\|107.007987900\|1087177\|1087177 | 0.016 | 0.24799 |
| 3B\|012.928798990\|1165422\|1165422 | 0.02 | 0.20062 | 2A\|043.555079010\|3222362\|3222362 | 0.017 | 0.19637 |
| 3D\|147.079710800\|1108739\|1108739 | 0.022 | 0.19969 | 6A\|067.991408960\|1237708\|1237708 | 0.024 | 0.22279 |
| 3A\|075.704509290\|1086515\|1086515 | 0.03 | 0.22183 | 6A\|028.951443700\|1094966\|1094966 | 0.027 | 0.21995 |
| 3B\|017.067952280\|998573\|998573 | 0.03 | 0.24508 | 2B\|033.737015920\|4733926\|4733926 | 0.027 | 0.24342 |
| 2B\|013.546408570\|977308\|977308 | 0.032 | 0.24293 | 3B\|029.893695730\|3222379\|3222379 | 0.029 | 0.18924 |
| 2B\|107.007987900\|1087177\|1087177 | 0.032 | 0.24322 | 3B\|011.134281090\|3027768\|3027768 | 0.029 | 0.24021 |
| 3B\|065.420680590\|1153055\|1153055 | 0.033 | 0.2198 | 2B\|012.351178520\|2297566\|2297566 | 0.031 | 0.23973 |
| 3A\|002.485631701\|3952392\|3952392 | 0.041 | 0.18783 | 2B\|057.230408490\|3064921\|3064921 | 0.036 | 0.21537 |
| 3A\|004.294450061\|4990595\|4990595 | 0.041 | 0.21675 | 1B\|037.446808880\|4991863\|4991863 | 0.036 | 0.21551 |
| 3B\|029.893695730\|3222379\|3222379 | 0.042 | 0.18517 | 2A\|104.293924100\|1110657\|1110657 | 0.038 | 0.18584 |
| 3B\|005.829269954\|2256562\|2256562 | 0.042 | 0.23713 | 6A\|048.226863160\|1150993\|1150993 | 0.038 | 0.21467 |
| 2A\|047.886253160\|5971468\|5971468 | 0.043 | 0.21221 | 7B\|097.452251360\|1126833\|1126833 | 0.041 | 0.18452 |
| 2B\|023.182120080\|1251215\|1251215 | 0.043 | 0.21221 | 7B\|100.338066000\|1096412\|1096412 | 0.041 | 0.18452 |
| 3B\|009.173872085\|1083589\|1083589 | 0.044 | 0.21107 | 7B\|100.338066000\|1276616\|1276616 | 0.041 | 0.18452 |
|  |  |  | 7B\|100.475730800\|3937689\|3937689 | 0.041 | 0.18452 |
|  |  |  | 2B\|012.351178520\|2291877\|2291877 | 0.041 | 0.21291 |
|  |  |  | 3B\|010.530635430\|4989647\|4989647 | 0.041 | 0.23513 |
|  |  |  | 2B\|026.939223010\|1093835\|1093835 | 0.044 | 0.20929 |

| **Drought-stressed** | | | **Non-stressed** | | |
| --- | --- | --- | --- | --- | --- |
| **Marker** | **Perm P** | **Marker R^2^** | **Marker** | **Perm P** | **Marker R^2^** |
| **Spike length** | | | | | |
| 3A\|070.942486970\|1104502\|1104502 | 0.005 | 0.19608 | 1B\|184.429245100\|1113389\|1113389 | 0.002 | 0.20749 |
| 2B\|107.007987900\|1087177\|1087177 | 0.005 | 0.23609 | 3A\|056.634055130\|3934533\|3934533 | 0.004 | 0.21891 |
| 2B\|107.234832300\|1159774\|1159774 | 0.006 | 0.18982 | 4B\|042.180040830\|1081624\|1081624 | 0.004 | 0.22577 |
| 5B\|118.415533200\|2303045\|2303045 | 0.008 | 0.21189 | 4B\|042.901192180\|1027953\|1027953 | 0.004 | 0.22577 |
| 2B\|106.869346000\|3023780\|3023780 | 0.009 | 0.18772 | 2B\|107.234832300\|1159774\|1159774 | 0.006 | 0.18905 |
| 1B\|176.095445600\|1259406\|1259406 | 0.01 | 0.18276 | 5B\|000.649324338\|1209883\|1209883 | 0.008 | 0.17696 |
| 7A\|065.934336980\|4541148\|4541148 | 0.01 | 0.18356 | 5D\|138.209637900\|7157166\|7157166 | 0.008 | 0.19944 |
| 5D\|019.203412160\|1161544\|1161544 | 0.01 | 0.18569 | 1B\|063.445873190\|3937163\|3937163 | 0.008 | 0.19985 |
| 3B\|011.134281090\|3027768\|3027768 | 0.011 | 0.22365 | 2B\|107.007987900\|1087177\|1087177 | 0.008 | 0.22003 |
| 2A\|060.600016560\|2361439\|2361439 | 0.017 | 0.17704 | 1B\|176.095445600\|1259406\|1259406 | 0.01 | 0.17524 |
| 3B\|027.228283330\|304361\|wPt-5432 | 0.017 | 0.17801 | 2A\|043.555079010\|3222362\|3222362 | 0.011 | 0.17382 |
| 2B\|106.857184700\|3533741\|3533741 | 0.017 | 0.20066 | 2A\|060.600016560\|2361439\|2361439 | 0.013 | 0.17187 |
| 3A\|137.708449300\|980006\|980006 | 0.019 | 0.17557 | 6A\|048.638907120\|3944784\|3944784 | 0.013 | 0.19444 |
| 5B\|000.649324338\|1209883\|1209883 | 0.025 | 0.17252 | 1D\|079.079510220\|1078020\|1078020 | 0.015 | 0.17008 |
| 1B\|045.390774230\|1294103\|1294103 | 0.03 | 0.19166 | 6B\|030.982252730\|1252863\|1252863 | 0.016 | 0.19243 |
| 6B\|031.043100140\|1237876\|1237876 | 0.031 | 0.16808 | 3A\|070.942486970\|1104502\|1104502 | 0.022 | 0.16746 |
| 5B\|114.361194300\|1126378\|1126378 | 0.032 | 0.20907 | 5B\|120.771719000\|2275671\|2275671 | 0.025 | 0.20819 |
| 1B\|043.312855080\|1162221\|1162221 | 0.034 | 0.16642 | 1D\|079.958633130\|3954634\|3954634 | 0.026 | 0.16481 |
| 1B\|044.758067030\|1266583\|1266583 | 0.034 | 0.16642 | 5B\|118.415533200\|2303045\|2303045 | 0.026 | 0.18742 |
| 1B\|045.177576690\|3022879\|3022879 | 0.034 | 0.16642 | 1D\|082.419690380\|1096857\|1096857 | 0.026 | 0.18855 |
| 1B\|046.175945760\|5325402\|5325402 | 0.034 | 0.16642 | 2B\|106.869346000\|3023780\|3023780 | 0.029 | 0.16331 |
| 1B\|047.111474150\|7346672\|7346672 | 0.034 | 0.16642 | 2A\|058.063556250\|1062330\|1062330 | 0.029 | 0.16357 |
| 1B\|047.741224110\|7334370\|7334370 | 0.034 | 0.16642 | 6B\|031.510249370\|4992590\|4992590 | 0.029 | 0.20528 |
| 1B\|051.254224490\|7346032\|7346032 | 0.034 | 0.16642 | 2B\|087.475859870\|3944716\|3944716 | 0.032 | 0.16243 |
| 1B\|051.289701790\|3950468\|3950468 | 0.034 | 0.16642 | 2B\|061.547753160\|4542690\|4542690 | 0.033 | 0.18528 |
| 1B\|054.040907460\|1266945\|1266945 | 0.034 | 0.16642 | 6A\|067.991408960\|1237708\|1237708 | 0.038 | 0.18309 |
| 1B\|061.689082610\|1122393\|1122393 | 0.034 | 0.16642 | 1D\|079.061658050\|5324336\|5324336 | 0.038 | 0.18326 |
| 1B\|061.689082610\|4261902\|4261902 | 0.034 | 0.16642 | 7B\|097.452251360\|1126833\|1126833 | 0.04 | 0.15854 |
| 1B\|063.445873190\|4005038\|4005038 | 0.034 | 0.16642 | 7B\|100.338066000\|1096412\|1096412 | 0.04 | 0.15854 |
| 1B\|066.188180470\|1164185\|1164185 | 0.034 | 0.16642 | 7B\|100.338066000\|1276616\|1276616 | 0.04 | 0.15854 |
| 1B\|070.209793990\|7345501\|7345501 | 0.034 | 0.16642 | 7B\|100.475730800\|3937689\|3937689 | 0.04 | 0.15854 |
| 1B\|070.891907290\|1232724\|1232724 | 0.034 | 0.16642 | 5D\|019.203412160\|1161544\|1161544 | 0.04 | 0.15888 |
| 1B\|247.531565800\|1699070\|1699070 | 0.034 | 0.16642 | 7A\|065.934336980\|4541148\|4541148 | 0.044 | 0.15726 |
| 1B\|263.257205600\|1767653\|1767653 | 0.034 | 0.16642 | 2B\|057.230408490\|3064921\|3064921 | 0.044 | 0.18076 |
| 2A\|004.227547215\|1732419\|1732419 | 0.034 | 0.16642 |  |  |  |
| 2A\|007.484441636\|1162329\|1162329 | 0.034 | 0.16642 |  |  |  |
| 3A\|056.634055130\|4539513\|4539513 | 0.034 | 0.16642 |  |  |  |
| 5A\|069.124959010\|993853\|993853 | 0.034 | 0.16642 |  |  |  |
| 6A\|086.477776400\|1238110\|1238110 | 0.034 | 0.16642 |  |  |  |
| 6B\|073.055837020\|978839\|978839 | 0.034 | 0.16642 |  |  |  |
| 7D\|013.562439360\|1216320\|1216320 | 0.034 | 0.16642 |  |  |  |
| 7D\|082.070949060\|1387325\|1387325 | 0.034 | 0.16642 |  |  |  |
| 6A\|067.991408960\|1237708\|1237708 | 0.034 | 0.18993 |  |  |  |
| 2B\|087.475859870\|3944716\|3944716 | 0.035 | 0.16606 |  |  |  |
| 4B\|042.901192180\|3954457\|3954457 | 0.035 | 0.18907 |  |  |  |
| 5B\|001.190695579\|1088009\|1088009 | 0.037 | 0.18818 |  |  |  |
| 5B\|120.771719000\|2275671\|2275671 | 0.037 | 0.20658 |  |  |  |
| 3A\|134.034465000\|3956339\|3956339 | 0.038 | 0.18774 |  |  |  |
| 6A\|048.638907120\|4406711\|4406711 | 0.044 | 0.20467 |  |  |  |

| **Drought-stressed** | | | **Non-stressed** | | |
| --- | --- | --- | --- | --- | --- |
| **Marker** | **Perm P** | **Marker R^2^** | **Marker** | **Perm P** | **Marker R^2^** |
| **Spikelet per spike** | | | | | |
| 6A\|028.951443700\|1094966\|1094966 | 0.003 | 0.31618 | 6B\|030.951829030\|1094565\|1094565 | 0.004 | 0.29618 |
| 3B\|027.228283330\|304361\|wPt-5432 | 0.004 | 0.27662 | 2B\|107.007987900\|1087177\|1087177 | 0.004 | 0.32519 |
| 6A\|048.638907120\|2281875\|2281875 | 0.004 | 0.3336 | 6B\|031.043100140\|1237876\|1237876 | 0.005 | 0.25697 |
| 5D\|019.203412160\|1161544\|1161544 | 0.006 | 0.26843 | 5B\|000.000000000\|3023157\|3023157 | 0.005 | 0.28927 |
| 2D\|126.901211300\|1109826\|1109826 | 0.007 | 0.25933 | 5A\|081.525617550\|4542594\|4542594 | 0.006 | 0.28448 |
| 3A\|134.034465000\|3945820\|3945820 | 0.007 | 0.28985 | 2D\|144.969480900\|1104828\|1104828 | 0.007 | 0.28341 |
| 1B\|063.445873190\|3937163\|3937163 | 0.007 | 0.29219 | 4B\|042.901192180\|3954457\|3954457 | 0.009 | 0.28057 |
| 1B\|176.095445600\|1259406\|1259406 | 0.009 | 0.25508 | 6B\|030.982252730\|1252863\|1252863 | 0.012 | 0.26518 |
| 3A\|004.294450061\|4990595\|4990595 | 0.009 | 0.28386 | 6A\|028.951443700\|1094966\|1094966 | 0.012 | 0.26633 |
| 6A\|049.559984320\|2280316\|2280316 | 0.009 | 0.30866 | 1D\|067.245066070\|4262641\|4262641 | 0.012 | 0.2701 |
| 3D\|147.079710800\|1108739\|1108739 | 0.01 | 0.2527 | 6B\|030.951829030\|1765837\|1765837 | 0.012 | 0.29597 |
| 2D\|144.969480900\|1104828\|1104828 | 0.013 | 0.27969 | 6A\|049.559984320\|2280316\|2280316 | 0.012 | 0.29643 |
| 4B\|042.901192180\|3954457\|3954457 | 0.017 | 0.27778 | 2D\|148.989565100\|374614\|wPt-4329 | 0.013 | 0.23457 |
| 6B\|078.748041400\|3958789\|3958789 | 0.018 | 0.30002 | 6B\|031.199477690\|4990947\|4990947 | 0.015 | 0.26184 |
| 3A\|056.634055130\|3934533\|3934533 | 0.024 | 0.27215 | 6B\|033.035441000\|1300029\|1300029 | 0.015 | 0.26184 |
| 1D\|067.245066070\|4262641\|4262641 | 0.029 | 0.26746 | 3A\|004.285602847\|3959705\|3959705 | 0.015 | 0.26207 |
| 2A\|091.748081010\|1117352\|1117352 | 0.037 | 0.26411 | 3A\|134.034465000\|3945820\|3945820 | 0.015 | 0.26406 |
| 3A\|004.285602847\|3959705\|3959705 | 0.037 | 0.26454 | 6B\|078.748041400\|3958789\|3958789 | 0.017 | 0.28445 |
| 3A\|076.019683050\|4394549\|4394549 | 0.038 | 0.23379 | 6D\|000.191508874\|5324047\|5324047 | 0.022 | 0.25655 |
| 3A\|002.485631701\|3952392\|3952392 | 0.04 | 0.23339 | 1D\|082.419690380\|1096857\|1096857 | 0.023 | 0.25596 |
| 5B\|117.097644100\|3950938\|3950938 | 0.043 | 0.23173 | 1D\|079.958633130\|3954634\|3954634 | 0.028 | 0.22285 |
|  |  |  | 2D\|126.901211300\|1109826\|1109826 | 0.028 | 0.2231 |
|  |  |  | 3A\|004.294450061\|4990595\|4990595 | 0.028 | 0.2522 |
|  |  |  | 1D\|079.417276640\|1094132\|1094132 | 0.029 | 0.25187 |
|  |  |  | 5D\|052.977153290\|3532978\|3532978 | 0.03 | 0.22179 |
|  |  |  | 3A\|004.732320657\|4989854\|4989854 | 0.031 | 0.21887 |
|  |  |  | 1D\|079.079510220\|1078020\|1078020 | 0.031 | 0.2197 |
|  |  |  | 2B\|101.018822200\|1113485\|1113485 | 0.031 | 0.24993 |
|  |  |  | 6B\|031.429488930\|1131748\|1131748 | 0.031 | 0.2742 |
|  |  |  | 6B\|027.222942130\|1090582\|1090582 | 0.032 | 0.27264 |
|  |  |  | 6B\|031.160976350\|1724555\|1724555 | 0.037 | 0.27002 |
|  |  |  | 6B\|031.087340250\|4398260\|4398260 | 0.039 | 0.26929 |
|  |  |  | 6A\|056.724637920\|4539672\|4539672 | 0.04 | 0.2147 |
|  |  |  | 5B\|117.097644100\|3950938\|3950938 | 0.042 | 0.21408 |
|  |  |  | 6B\|035.712467660\|4989379\|4989379 | 0.042 | 0.24415 |
| **Kernels per spike** | | | | | |
| 6B\|079.586479380\|3949288\|3949288 | 0.016 | 0.23815 | 6B\|030.951829030\|1144567\|1144567 | 0.002 | 0.26852 |
| 7D\|160.711960400\|2358656\|2358656 | 0.017 | 0.20662 | 2D\|153.055365100\|1237263\|1237263 | 0.003 | 0.23728 |
| 2D\|153.055365100\|1237263\|1237263 | 0.017 | 0.20691 | 5B\|117.097644100\|3950938\|3950938 | 0.003 | 0.23779 |
| 6B\|030.951829030\|1144567\|1144567 | 0.017 | 0.23637 | 6B\|030.951829030\|1765837\|1765837 | 0.004 | 0.2811 |
| 2D\|148.989565100\|374614\|wPt-4329 | 0.019 | 0.20373 | 6B\|030.982252730\|1252863\|1252863 | 0.005 | 0.25133 |
| 1D\|058.851037050\|5411762\|5411762 | 0.021 | 0.23176 | 6B\|031.197452460\|3939783\|3939783 | 0.005 | 0.27574 |
| 6B\|029.393612280\|1003850\|1003850 | 0.022 | 0.25482 | 6B\|029.393612280\|1003850\|1003850 | 0.006 | 0.27067 |
| 6B\|031.822757590\|4540541\|4540541 | 0.025 | 0.25203 | 6B\|031.301254040\|2276412\|2276412 | 0.007 | 0.2463 |
| 6B\|031.043100140\|1237876\|1237876 | 0.029 | 0.19792 | 1D\|058.851037050\|5411762\|5411762 | 0.008 | 0.23862 |
| 5B\|000.000000000\|3023157\|3023157 | 0.036 | 0.2251 | 1B\|239.642526900\|1249348\|1249348 | 0.008 | 0.24518 |
| 6B\|026.564442100\|1115276\|1115276 | 0.039 | 0.24743 | 6B\|031.822757590\|4540541\|4540541 | 0.008 | 0.26855 |
| 5B\|001.190695579\|1088009\|1088009 | 0.044 | 0.22238 | 6B\|031.993678630\|1234486\|1234486 | 0.009 | 0.26076 |

| **Drought-stressed** | | | **Non-stressed** | | |
| --- | --- | --- | --- | --- | --- |
| **Marker** | **Perm P** | **Marker R^2^** | **Marker** | **Perm P** | **Marker R^2^** |
| **Kernels per spike** | | | | | |
|  |  |  | 2D\|148.923115000\|1124930\|1124930 | 0.012 | 0.20103 |
|  |  |  | 2D\|149.648499200\|1117423\|1117423 | 0.012 | 0.20103 |
|  |  |  | 2D\|149.851806300\|1260378\|1260378 | 0.012 | 0.20103 |
|  |  |  | 2D\|150.373883300\|1132957\|1132957 | 0.012 | 0.20103 |
|  |  |  | 6B\|079.586479380\|3949288\|3949288 | 0.012 | 0.23068 |
|  |  |  | 4B\|042.180040830\|1081624\|1081624 | 0.012 | 0.23374 |
|  |  |  | 4B\|042.901192180\|1027953\|1027953 | 0.012 | 0.23374 |
|  |  |  | 1D\|079.417276640\|1094132\|1094132 | 0.012 | 0.23376 |
|  |  |  | 6A\|049.559984320\|2280316\|2280316 | 0.012 | 0.25411 |
|  |  |  | 6B\|021.333495120\|1091969\|1091969 | 0.012 | 0.2551 |
|  |  |  | 6B\|026.564442100\|1115276\|1115276 | 0.012 | 0.25656 |
|  |  |  | 1A\|080.925753810\|3938842\|3938842 | 0.012 | 0.25695 |
|  |  |  | 2A\|127.024087100\|3943270\|3943270 | 0.014 | 0.197 |
|  |  |  | 2A\|123.580916200\|3949672\|3949672 | 0.014 | 0.22699 |
|  |  |  | 7A\|112.746111900\|5331823\|5331823 | 0.014 | 0.22699 |
|  |  |  | 5B\|000.000000000\|3023157\|3023157 | 0.014 | 0.22718 |
|  |  |  | 6B\|030.951829030\|2322413\|2322413 | 0.023 | 0.24512 |
|  |  |  | 1D\|079.417276640\|1039789\|1039789 | 0.024 | 0.22137 |
|  |  |  | 7B\|042.947028100\|986776\|986776 | 0.025 | 0.21966 |
|  |  |  | 6B\|023.023153150\|1154773\|1154773 | 0.028 | 0.2423 |
|  |  |  | 2D\|152.244494700\|1104321\|1104321 | 0.03 | 0.21798 |
|  |  |  | 2D\|155.591825400\|1279862\|1279862 | 0.034 | 0.21645 |
|  |  |  | 6B\|077.148647860\|2279482\|2279482 | 0.034 | 0.2399 |
|  |  |  | 6B\|030.951829030\|1139022\|1139022 | 0.038 | 0.23815 |
|  |  |  | 6B\|029.393612280\|1003850\|1003850 | 0.039 | 0.23789 |
|  |  |  | 2D\|148.923115000\|1096024\|1096024 | 0.04 | 0.18498 |
|  |  |  | 2D\|150.373883300\|1122467\|1122467 | 0.04 | 0.18498 |
|  |  |  | 6B\|041.061995810\|2309137\|2309137 | 0.042 | 0.21383 |

Perm P = Probability value; R^2^ = marker-trait correlation
